# Effects of remotely-delivered cognitive behavioral therapy for insomnia in type 2 diabetes: a randomized controlled trial

**DOI:** 10.1007/s11325-025-03469-y

**Published:** 2025-10-20

**Authors:** Similan Kirisri, Sirimon Reutrakul, Chutintorn Sriphrapradang, Saratcha Tiensuntisook, Naricha Chirakalwasan, Sunee Saetung, Chanatpon Aonnuam, Chatvara Areevut, Ratanaporn Jerawatana, Jarturong Siritienthong

**Affiliations:** 1 Division of Endocrinology and Metabolism, Department of Medicine, Faculty of Medicine Ramathibodi Hospital, Mahidol University, Bangkok, Thailand; 2 Division of Endocrinology, Diabetes and Metabolism University of Illinois Chicago, Chicago, IL USA; 3 Faculty of Medicine Ramathibodi Hospital , Chakri Naruebodindra Medical Institute, Samut Prakan, Thailand; 4 Division of Pulmonary and Critical Care Medicine, Department of Medicine, Faculty of Medicine, Chulalongkorn University, Bangkok, Thailand; 5 Excellence Center For Sleep Disorders, King Chulalongkorn Memorial Hospital, Thai Red Cross Society, Bangkok, Thailand; 6 Department of Clinical Epidemiology and Biostatistics, Faculty of Medicine Ramathibodi Hospital, Mahidol University, Bangkok, Thailand; 7 Division of Nursing, Faculty of Ramathibodi Hospital, Mahidol University, Bangkok, Thailand; 8 Department of Psychiatry, Faculty of Medicine Ramathibodi Hospital, Mahidol University, Bangkok, Thailand; 9 Ramathibodi Sleep Disorders Center, Faculty of Medicine Ramathibodi Hospital, Mahidol University, Bangkok, Thailand

**Keywords:** Cognitive behavioral therapy, Insomnia, Sleep quality, Type 2 diabetes, Glucose

## Abstract

**Purpose:**

To evaluate the effects of remotely delivered cognitive behavioral therapy for insomnia (CBTI) on subjective sleep quality, glycemic control, and objective sleep parameters in individuals with type 2 diabetes (T2D) and insomnia.

**Methods:**

Forty adults with non-insulin-treated T2D and insomnia were randomized to CBTI (*n* = 20) or health education (HE, *n* = 20), delivered weekly via one-hour online sessions for eight weeks. The primary outcome was self-reported sleep quality (Pittsburgh Sleep Quality Index, PSQI). Secondary outcomes included actigraphy-based sleep measures, glycemic control (A1C, fasting glucose), insomnia symptoms, anxiety, depression, and quality of Life. Data were collected at baseline, week 8, and week 16. Mixed-effects linear regression was used to assess between-group differences.

**Results:**

At week 8, no significant difference in PSQI was observed between groups, but the CBTI group showed improved actigraphy-based sleep regularity (variation of sleep duration), mean difference − 21.84 min (95% CI -41.64, -2.05; *P* = 0.031). At week 16, CBTI led to a greater reduction in anxiety symptoms (*P* = 0.039). There were no differences in other outcomes. In per-protocol analysis (CBTI: *n* = 15; HE: *n* = 10), CBTI resulted in improved subjective sleep quality (*P* = 0.042), sleep regularity (*P* = 0.018) and fasting glucose at week 8 (mean difference − 34.27 mg/dL; 95% CI -55.16, -13.37; *P* = 0.001). Satisfaction was high in both groups.

**Conclusion:**

CBTI improved sleep regularity and anxiety in T2D patients with insomnia. Adherence to CBTI also led to fasting glucose reductions, supporting its role in glycemic management. Sleep-focused interventions like CBTI should be integrated into care for T2D with insomnia to optimize sleep and metabolic outcomes.

**Supplementary Information:**

The online version contains supplementary material available at 10.1007/s11325-025-03469-y.

## Introduction

Sleep health is now recognized as a critical component of management in persons with prediabetes and diabetes. In 2025, The American Diabetes Association places sleep on the level playing field as other important lifestyle behaviors [1]. One thirds of persons with type 2 diabetes (T2D) experienced poor sleep including difficulties falling or staying asleep [2]. Poor sleep quality in persons with diabetes is associated with suboptimal glycemic control [3] and poor quality of life [4].

Cognitive Behavioral Therapy for Insomnia (CBTI) is a standard non-pharmacological treatment for insomnia [5]. This therapy involves identifying and addressing thoughts, beliefs, and behaviors that contribute to insomnia [5, 6]. CBTI, both delivered in-person or remotely, has been shown to improve sleep onset, sleep latency, sleep efficiency, and reduce wake time after sleep onset (WASO) [7, 8]. To date, only a few studies have explored the effects of CBTI on glycemic control specifically in persons with T2D and insomnia [9, 10]. Alshehri et al. randomized 28 participants with T2D to 6-week in-person CBTI or health education [10]. There was a reduction in hemoglobin A1C (A1C) in the CBTI group of 0.5%, along with reduced fatigue and improved self-care behavior, while there was no change in the health education group [10]. In another study, 187 participants with T2D and poor sleep quality were randomized to 7-week group sessions consisted of CBT with aerobic exercise plus usual care or usual care alone [9]. The CBTI group had significantly improved sleep quality and quality of life, along with a lower A1C by 0.89% at 6-month follow up as compared to the usual care group [9]. While these data were promising, sleep was mainly subjectively assessed, and most sessions were conducted in-person. Therefore, the knowledge gap remains if CBTI in persons with T2D would lead to improvement in glycemic control and changes in objectively measured sleep parameters. Whether the remotely conducted CBTI in persons with T2D is effective has not been widely tested.

This open-labelled randomized study aimed to evaluate the effects of a remotely-delivered CBTI in persons with T2D and insomnia as compared to health education. The primary outcome was self-reported sleep quality, assessed by the Pittsburgh Sleep Quality Index (PSQI) [11, 12]. Secondary outcomes included glycemic and inflammatory markers, objective sleep parameters, and other patient-reported outcomes. We hypothesized that the CBTI would improve both sleep and glycemic control in adults with T2D and insomnia. The results of the study would inform clinicians on the management of insomnia in persons with T2D.

## Method

### Study design

This study was an open-labelled randomized controlled trial (November 2023 to October 2024). Eligible participants were randomly assigned to either the CBTI group or the health education (control) group using a computer-generated random number table. Randomization was performed by S.R. who had no direct contact with participants and was concealed until baseline assessment was complete.

## Participants

We included adults aged 30–65 years with T2D with insomnia symptoms defined as a score ≥ 15 on the Thai version of Insomnia Severity Index (ISI) [13] who were being followed at the outpatient clinics, Faculty of Medicine Ramathibodi Hospital, Bangkok. Inclusion criteria were (1) A1C levels of 6.5–9.9% on a stable regimen of oral diabetes medication for at least three months; (2) had access to a computer or tablet with a stable internet connection to participate in online sessions and were committed to attending all eight study sessions; (3) if sleep aids or other medications affecting sleep were used, these had to remain at the same type and dosage for at least three months. Exclusion criteria were night shift work, recent severe hypoglycemia or diabetic ketoacidosis, significant medical comorbidities, symptoms suggestive of obstructive sleep apnea which were untreated (STOP-BANG score ≥ 5) [14], active psychiatric disorders, pregnancy or breastfeeding or nighttime caregiving for others, and significant alcohol (≥ 15 drinks/week for men or ≥ 8 for women) or tobacco use.

This study was conducted in accordance with the ethical standard as laid down in the 1964 Declaration of Helsinki, approved by the Human Research Ethics Committee, Faculty of Medicine Ramathibodi Hospital, Mahidol University (MURA2023/773), and registered at clinicaltrials.gov (NCT06202742). All participants provided written informed consent.

After enrollment (by S.K.), participants were interviewed regarding their medical history, diabetes duration, and diabetic complications. Medication use and comorbidities were reviewed from the medical records. Weight (kg) and height (cm) were measured and body mass index (kg/m^2^) was calculated.

## Intervention

Eight weekly one-hour online sessions, were delivered via the Zoom platform for both groups.

## Cognitive behavioral therapy for insomnia (CBTI)

The CBTI protocol was designed and conducted by psychiatrists (J.S. and S.T.) and covered the CBTI core therapeutic techniques [5, 6]. A sleep diary was utilized to monitor sleep patterns, identify issues, and guide interventions. Details of each week intervention are outlined below.

### Week 1

CBTI framework, focusing on understanding sleep mechanisms and factors influencing sleep, was introduced. Sleep hygiene education was provided. Participants were guided to identify and challenge false beliefs about sleep.

### Week 2

Discussing sleep problems from the past week and review of participants’ sleep diaries. Stimulus control therapy [5, 6, 15] was introduced. Participants were advised to go to bed only when sleepy, avoid non-sleep activities in bed, leave the bed if unable to sleep within 15–20 min, maintain a consistent wake time, and avoid daytime napping [6]. Sleep efficiency (percentage of time in bed spent sleeping) was calculated. Sleep restriction [5, 6, 15] therapy was also initiated: time in bed (TIB) was restricted to match the average total sleep time (TST) derived from the baseline sleep diary.

### Week 3

Sleep diaries were again reviewed at the beginning of the session, and individualized sleep prescriptions were adjusted. For participants with improved sleep efficiency, TIB was extended by 15 min; for those with persistently low efficiency, further restriction was applied. Relaxation techniques and stress management strategies [5, 6, 15] were incorporated. These techniques were practiced during sessions and encouraged to be used at home.

### Week 4

The process of adjusting sleep prescriptions continued. The rationale and calculation methods of sleep restriction were explained in detail, with practical examples drawn from participants’ own diaries to enhance understanding and adherence. Relaxation training was reinforced.

### Week 5–7

Cognitive therapy [5, 6, 15] was utilized to challenge and modify maladaptive thoughts about sleep, along with integrating relaxation techniques and preparing participants for relapse prevention.

### Week 8

The intervention was summarized, key strategies were reinforced, and guidance was provided on relapse prevention.

## Health education (HE)

General health education was provided to the HE group, excluding topics related to sleep quality or glycemic control. The topics covered adult vaccinations, osteoporosis, cancer screening, tuberculosis, dyspepsia, cerebrovascular disease, coronary syndrome and chronic kidney disease.

### Outcomes

Assessments were conducted at three time points: baseline, week 8, and week 16 (8 weeks post-intervention).

#### The primary outcome

was self-reported sleep quality as assessed by the Pittsburgh Sleep Quality Index (PSQI) [11, 12] at week 8. PSQI assessed sleep quality across seven dimensions including subjective sleep quality, sleep duration, sleep latency, sleep disturbances, daytime dysfunction, and sleep efficiency and the use of sleep medications. The total scores range from 0 to 21, with higher scores indicating poorer sleep quality [11, 12]. Subjective sleep quality dimension (question 6 of the PSQI) was also reported.

#### Secondary outcomes

Included the following:

- **Glycemic parameters** (fasting glucose, A1C, insulin levels), and an inflammatory marker (high sensitivity C-reactive protein, hs-CRP) were assessed at baseline and week 8. The assays were performed at the clinical laboratory, Faculty of Medicine Ramathibodi Hospital. Fasting glucose assays were performed using a hexokinase/glucose-6-phosphate dehydrogenase method. A1C, insulin and hs-CRP assays were performed using a Turbidimetric inhibition immunoassay method, a chemiluminescence method, and a nephelometry immunoassay method, respectively.
- **Objective sleep monitoring** was assessed using an Actiwatch 2 or Actiwatch Spectrum for 7 days at baseline and week 8. Using sleep diary, event markers, light and activity signals, the researcher derived bedtime and wake time [16]. Sleep parameters were analyzed using Philips Actiware software. Sleep onset and offset, sleep duration (hours), sleep efficiency (percentage of time in bed spent sleeping), sleep latency and wake time after sleep onset (WASO) were derived. The sleep midpoint was defined as the midpoint between sleep onset and offset. Sleep regularity was assessed using the standard deviation (SD) of sleep duration across recording nights, with lower number indicating more regular sleep. Sleep regularity is recognized as one of the multidimensional sleep health which contributes to cardiometabolic health, and can be measured in several ways including SD of sleep timing or duration [17]. In this study, we chose SD of sleep duration as this was previously shown to be associated with glycemic control in people with diabetes [18].
- **Patient reported outcomes** were assessed using validated questionnaires. The Insomnia Severity Index (ISI) assessed insomnia severity with scores ranging from 0 to 28, with higher scores indicating greater insomnia symptoms [13, 19]. The Epworth Sleepiness Scale (ESS) assessed daytime sleepiness with scores ranging from 0 to 24; higher scores reflecting greater sleepiness [20]. The Generalized Anxiety Disorder Scale (GAD-7) measured anxiety severity with scores ranging from 0 to 21,; higher scores indicating greater anxiety [21]. Patient Health Questionnaire-9 (PHQ-9) assessed depressive symptoms, yielding a total score of 0–27 with higher scores indicating greater depression [22]. The Thai version of the Perceived Stress Scale-10 (T-PSS-10) assessed perceived stress levels, with higher scores indicating greater stress [23]. The WHOQOL-BREF-THAI assessed quality of Life with total scores ranging from 26 to 130; higher scores reflecting better quality of life [24].

## Adherence and program satisfaction

Adherence to the program was recorded. Program satisfaction was assessed in both groups at week 16 using a 0–5 Likert scale.

### Sample size

Sample size was calculated based on findings of PSQI score in persons with T2D and insomnia of 11.0 (2.5) [9], and CBTI in T2D with insomnia led to a reduction in PSQI score of 3.03 [9]. Randomizing 40 patients to the two treatment groups would have 80% power to detect a coefficient in PSQI of 2.27 with a two-sided alpha of 0.05 significance level.

### Statistical analysis

Statistical analyses were performed using Stata/SE 18.0. Normality was assessed using QQ-plots. For normally distributed baseline variables, means and standard deviations (SD) were calculated, while medians and interquartile ranges (IQR) were used for non-normally distributed variables. Categorical variables were presented as frequency and percentages.

Outcomes for each group were reported as mean with standard errors (SE). Between-group differences of changes from baseline (CBTI- HE) were analyzed using multilevel mixed-effects linear regression and reported as mean difference (MD) with 95% confidence intervals (95% CI). The minimum level of statistical significance was *P* < 0.05. A per-protocol analysis was conducted on participants who completed all eight sessions of the intervention.

## Results

**Fig. 1:**
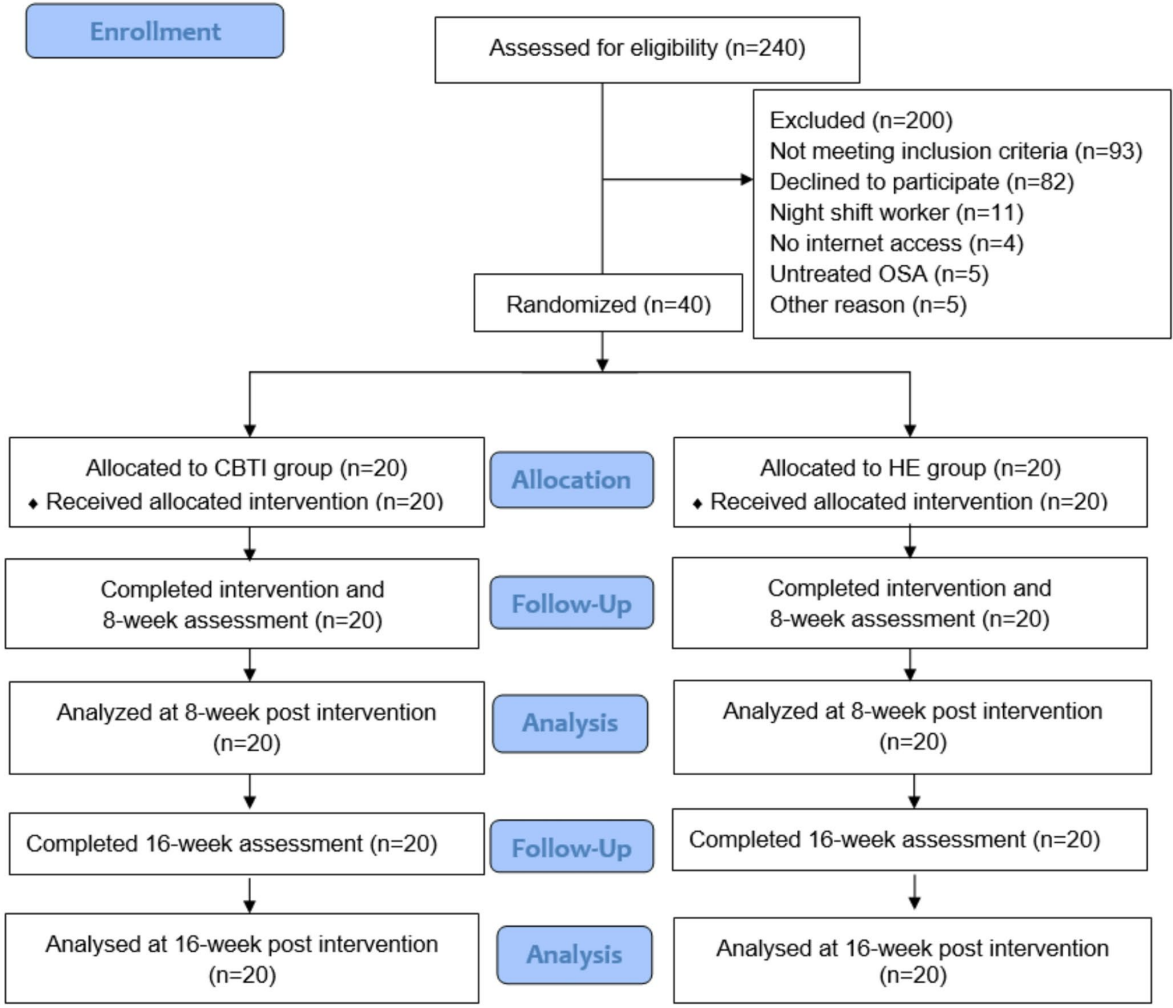
Flowchart of participant’s disposition at enrollment, allocation, followed – up and analysis according to CONSORT flow diagram

The flow of the study is shown in Fig. 1. A total of 240 T2D were screened for eligibility. After excluding 200 potential participants, forty eligible participants were enrolled and randomly allocated to the CBTI group (*n* = 20) or the HE group (*n* = 20). The demographic characteristics are summarized in Table 1. Mean age, sex distribution, mean BMI, diabetes duration, diabetic complications, comorbidities, and diabetes medication use were similar between the two groups. Insomnia symptoms and baseline glycemic control, and sleep characteristics (Table 1) were also similar. One participant in the CBTI group experienced a malfunctioned actiwatch, therefore the data was not available at baseline. Sleep medication use (melatonin, lorazepam and trazodone) was reported by 10% of participants in both groups.

**Table 1:** Baseline demographic and clinical characteristics of the participants

Characteristics	CBTI (*n* = 20)	Control (*n* = 20)	*P* value
Age (y), mean (SD)	55.7(6.10)	52.1 (10.23)	0.186
Female, n (%)	13(65)	14(70)	0.736
BMI (kg/m2)	29.17 (5.03)	30.95 (5.75)	0.303
Diabetes duration (y), median (IQR)	9.5 (6.5,15)	8 (4,15)	0.721
Age onset of DM (y), mean (SD)	45.4 (6.06)	42.8 (9.87)	0.323
Comorbidity, n (%)			
Hypertension	10 (50)	10 (50)	1.000
Dyslipidemia	19 (95)	19 (95)	1.000
Hypothyroidism	2 (10)	0 (0)	0.487
Obstructive sleep apnea	1 (5)	0 (0)	1.000
Chronic kidney disease	1 (5)	0 (0)	1.000
Asthma	1 (5)	1 (5)	1.000
Cerebrovascular disease	1 (5)	0 (0)	1.000
Diabetic complications			
Diabetic retinopathy, n (%)^a^	2 (10)	2 (10)	0.410
Diabetic kidney disease, n (%)^b^	4 (20)	7 (35)	0.361
Diabetes medications, n (%)			
Metformin	19 (95)	19 (95)	1.000
Sulfonylurea	10 (50)	9 (45)	0.752
Thiazolidinedione	5 (25)	8 (40)	0.311
DPP-4 inhibitor	11 (55)	7 (35)	0.204
SGLT-2 inhibitor	3 (15)	5 (25)	0.695
GLP-1 RA	3 (15)	2 (10)	1.000
Sleep medication use, n (%)	2 (10)	2 (10)	1.000
Time from blood test to intervention (days), mean (SD)	37.55 (15.06)	40.65 (24.27)	0.631
Insomnia severity index, mean (SD)	16.30 (2.08)	16.05 (1.67)	0.346
Self-reported sleep parameters			
PSQI score, mean (SE)	10 (0.65)	10.55 (0.65)	0.551
Self-reported sleep quality, mean, (SE)	1.6 (0.14)	1.55 (0.14)	0.802
Self-reported sleep latency (minutes), mean, (SE)	37 (3.75)	31 (3.75)	0.258
Self-reported sleep duration (h), mean (SE)	5.48 (0.21)	4.98 (0.21)	0.107
Self-reported sleep efficiency (%), mean (SE)	75 (3)	72 (3)	0.480
Actigraphy-derived sleep parameters ^c^			
Time in bed (h), mean (SE)	7.43 (0.19)	7.06(0.19)	0.164
Sleep duration (h), mean (SE)	6.24 (0.19)	5.76 (0.18)	0.068
Sleep efficiency (%), mean (SE)	84.30 (1.71)	81.62 (1.69)	0.265
WASO (min), mean (SE)	41.79 (3.77)	42.77 (3.72)	0.853
Sleep latency (min), mean (SE)	14.85 (3.17)	19.71 (3.11)	0.275
Sleep midpoint (hh: mm), mean (SE)	02:37 (00:14)	02:03 (00:14)	0.082
Sleep regularity (SD of sleep duration) (min), mean (SE)	61.57 (6.22)	55.10 (6.07)	0.457
Metabolic parameters			
Fasting glucose (mg/dL), mean (SE)	143.85 (6.63)	142.8 (6.63)	0.911
A1C (%), mean (SE)	7.65	7.33 (0.20)	0.252
	(0.20)		
Insulin (uIU/mL), mean (SE)	11.79 (1.78)	8.86 (1.78)	0.243
hs-CRP (mg/L), mean (SE)	3.90 (1.28)	1.82 (1.28)	0.251
Other Sleep and Psychological Questionnaire			
ESS, mean (SE)	7.6 (0.89)	8.25 (0.89)	0.605
GAD-7, mean (SE)	6.45 (0.66)	4.85 (0.66)	0.088
PHQ-9, mean (SE)	7.5 (0.80)	8.8 (0.80)	0.253
T-PSS-10, mean (SE)	14.7 (1.10)	16.8 (1.10)	0.179
WHOQOL-BREF-THAI, mean (SE)	90.85 (2.28)	89.75 (2.28)	0.733

*DPP-4inh* Dipeptidyl peptidase-4 inhibitor, *SGLT-2 inhibitor* Sodium-glucose cotransporter-2 inhibitor, *GLP-1 RA* Glucagon-like peptide-1 receptor agonist, *ISI* Insomnia Severity Index, *GAD-7* Generalized Anxiety Disorder Scale, *PHQ-9* Patient Health Questionnaire-9, *T-PSS-10* The Thai version of the Perceived Stress Scale-10, *WHOQOL-BREF-THAI* A Thai-language version of the WHOQOL-BREF, *ESS* The Epworth Sleepiness Scale

^a^*n*=35, ^b^*n*=34,^c^*n*=19 for CBTI group

In the CBTI group, 15 participants (75%) attended all 8 sessions, 4 (20%) attended 7 sessions and 1 (5%) attended 6 sessions. In the HE group, 10 participants (50%) attended all 8 sessions, 8 (40%) attended 7 sessions, 1 (5%) attended 6 sessions and 1 (5%) attended 5 sessions.

### Primary outcome

#### Self-reported sleep parameters

There were no significant differences in PSQI scores in the CBTI as compared to the HE group (Table 2). The mean differences (MD) were − 0.65 (95% CI: −2.79, 1.49, *P* = 0.552) at week 8, and − 0.25 (95% CI: −3.28, 2.78, *P* = 0.871) at week 16. In addition, PSQI subscales (self-reported sleep quality, sleep latency, duration, and efficiency) did not differ between groups.

**Table 2:** Self-reported and actigraphy-derived sleep parameters at baseline, week 8 and week 16 (*N* = 40)

Response variables	CBTI (*n* = 20)			HE (*n* = 20)			Between-group difference in change from baseline (CBTI- HE)			
							Week 8 vs. baseline		Week 16 vs. baseline	
	Baseline	Week 8	Week 16	Baseline	Week 8	Week 16	MD (95%CI)	*P* value	MD (95%CI)	*P* value
Self-reported sleep parameters										
PSQI score	10 (0.65)	6.5 (0.65)	6.35 (0.65)	10.55 (0.65)	7.7 (0.65)	7.15 (0.65)	−0.65 (−2.79, 1.49)	0.552	−0.25 (−3.28, 2.78)	0.871
Self-reported sleep quality	1.6 (0.14)	1.05 (0.14)	1.05 (0.14)	1.55 (0.14)	1.2 (0.14)	1.5 (0.14)	−0.2 (−0.68, 0.28)	0.414	−0.50 (−1.18, 0.18)	0.149
Self-reported sleep latency (minutes)	37 (3.75)	22.75 (3.75)	19.5 (3.75)	31 (3.75)	18.5 (3.75)	14.2 (3.75)	−1.75 (−14.26,10.76)	0.784	−0.7 (−18.39, 16.99)	0.938
Self-reported sleep duration (h)	5.48 (0.21)	6.15 (0.21)	6.1 (0.21)	4.98 (0.21)	5.5 (0.21)	5.73 (0.21)	0.15 (−0.51, 0.81)	0.657	−0.13 (−1.06, 0.81)	0.794
Self-reported sleep efficiency (%)	75 (3)	86 (3)	84 (3)	72 (3)	81(3)	85 (3)	2 (−10, 13)	0.780	−4 (−20, 12)	0.626
Actigraphy-derived sleep parameters*										
Time in bed (h)	7.43 (0.19)	7.36 (0.19)	-	7.06(0.19)	7.09 (0.19)	-	−0.06 (−0.53, 0.41)	0.808	-	-
Sleep duration (h)	6.24 (0.19)	6.21 (0.18)	-	5.76 (0.18)	5.71 (0.18)	-	0.06 (−0.47, 0.58)	0.830	-	-
Sleep efficiency (%)	84.30 (1.71)	84.44 (1.69)	-	81.62 (1.69)	80.69 (1.69)	-	0.99 (−3.21, 5.21)	0.642	-	-
WASO (min)	41.79 (3.77)	40.87 (3.72)	-	42.77 (3.72)	36.82 (3.72)	-	5.37 (−2.19, 12.94)	0.164	-	-
Sleep latency (min)	14.85 (3.17)	18.27 (3.11)	-	19.71 (3.11)	22.94 (3.11)	-	0.30 (−7.82, 8.42)	0.943	-	-
Sleep midpoint (hh: mm)	02:37 (00:14)	02:38 (00:14)	-	02:03 (00:14)	02:07 (00:14)	-	−5.0 min (44, 34)	0.804	-	-
Sleep regularity (SD of sleep duration) (min)	61.57 (6.22)	54.71 (6.07)	-	55.10 (6.07)	68.25 (6.07)	-	−21.84 (−41.64, −2.05)	0.031	-	-

Values are presented as mean (standard error). Between-group differences represent mean differences (MD) with 95% confidence intervals

*n = 19 for CBTI group at baseline

### Secondary outcomes

#### Objective sleep parameters

At week 8, the CBTI group showed a significant improvement in sleep regularity compared to the HE group, MD −21.84 min (95% CI: −41.64, −2.05, *P* = 0.031), Table 2. There were no significant differences between groups in time in bed, sleep duration, sleep efficiency, WASO, sleep latency, or sleep midpoint.

### Metabolic parameters

At week 8, the CBTI group exhibited a trend toward lower fasting glucose levels compared to the HE group, though the difference was not statistically significant, MD −15.45 mg/dL, (95% CI: −32.55, 1.65, *P* = 0.077), Table 3. No significant differences were observed between the groups in A1C, insulin or hs-CRP levels.

**Table 3:** Distribution of metabolic parameters and sleep/psychological questionnaires at baseline, week 8 and week 16 (*N* = 40)

Response Variables	CBTI (*n* = 20)			HE (*n* = 20)			Between-group difference in change from baseline (CBTI- HE)			
							Week 8 vs. baseline		Week 16 vs. baseline	
	Baseline	Week 8	Week 16	Baseline	Week 8	Week 16	MD (95%CI)	*P* value	MD (95%CI)	*P* value
Metabolic parameters										
Fasting glucose (mg/dL)	143.85 (6.63)	132.8 (6.63)	-	142.8 (6.63)	147.2 (6.63)	-	−15.45 (−32.55,1.65)	0.077	-	-
A1C (%)	7.65 (0.20)	7.30 (0.20)	-	7.33 (0.20)	7.36 (0.20)	-	−0.37 (−0.90, 0.15)	0.164	-	-
Insulin (uIU/mL)	11.79 (1.78)	12.00 (1.78)	-	8.86 (1.78)	8.03 (1.78)	-	1.03 (−0.54, 2.59)	0.198	-	-
hs-CRP (mg/L)	3.90 (1.28)	5.11 (1.28)	-	1.82 (1.28)	3.53 (1.28)	-	−0.50 (−3.17, 2.18)	0.716	-	-
Sleep and Psychological Questionnaires										
ISI	16.3 (0.78)	11.15 (0.78)	7.35 (0.78)	16.05 (0.78)	11.3 (0.78)	9.55 (0.78)	−0.4 (−3.25, 2.45)	0.783	−2.45 (−6.48, 1.58)	0.233
ESS	7.6 (0.89)	8.05 (0.89)	5.35 (0.89)	8.25 (0.89)	6.05 (0.89)	7.75 (0.89)	2.65 (−0.67, 5.97)	0.118	−1.75 (−6.45, 2.95)	0.465
GAD-7	6.45 (0.66)	4.0 (0.66)	3.4 (0.66)	4.85 (0.66)	4.1 (0.66)	4.35 (0.66)	−1.7 (−3.41, 0.01)	0.052	−2.55 (−4.97, −0.13)	0.039
PHQ-9	7.5 (0.80)	5.25 (0.80)	4.75 (0.80)	8.8 (0.80)	6.2 (0.80)	5.75 (0.80)	0.35 (−1.92, 2.62)	0.762	0.3 (−2.91, 3.51)	0.855
T-PSS-10	14.7 (1.10)	13.15 (1.10)	13.05 (1.10)	16.8 (1.10)	14.05 (1.10)	12.65 (1.10)	1.2 (−2.12, 4.52)	0.478	2.5 (−2.19, 7.19)	0.296
WHOQOL-BREF-THAI	90.85 (2.28)	97.9 (2.28)	98.5 (2.28)	89.75 (2.28)	92.2 (2.28)	95.3 (2.28)	4.6 (−1.97, 11.17)	0.170	2.1 (−7.19, 11.39)	0.658

Values are presented as mean (standard error). Between-group differences represent mean differences (MD) with 95% confidence intervals

*ISI* Insomnia Severity Index, *GAD-7* Generalized Anxiety Disorder Scale, *PHQ-9* Patient Health Questionnaire-9, *T-PSS-10* The Thai version of the Perceived Stress Scale-10, *WHOQOL-BREF-THAI* A Thai-language version of the WHOQOL-BREF, *ESS* The Epworth Sleepiness Scale

### Patient reported-outcomes

At week 16, the CBTI group showed significant greater reduction in anxiety scores compared to the HE group, MD −2.55 (95% CI: −4.97, −0.13, *P* = 0.039), Table 3. Insomnia symptoms, daytime sleepiness, depressive symptoms, stress and quality of life, however, did not differ between groups.

### Per-protocol analysis

The per-protocol analysis, which included only participants who completed all eight sessions of CBTI (*n* = 15) or HE (*n* = 10), was conducted. The PSQI scores showed no significant differences between the groups, Table 4. However, at week 16, self-reported sleep quality significantly (item 6 of PSQI) improved in the CBTI group as compared to the HE group, MD −0.9 (95% CI: −1.77, −0.03, *P* = 0.042). In addition, sleep regularity as assessed by actigraphy, showed a significant improvement in the CBTI group at week 8, MD-31.69 min (95% CI: −57.92, −5.47, *P* = 0.018).

**Table 4:** Per-protocol analysis of self-reported and actigraphy-derived sleep parameters at baseline, week 8 and week 16 (*N* = 25)

Response variables	CBTI (*n* = 15)			HE (*n* = 10)			Between-group difference in change from baseline (CBTI- HE)			
							Week 8 vs. baseline		Week 16 vs. baseline	
	Baseline	Week 8	Week 16	Baseline	Week 8	Week 16	MD (95%CI)	*P* value	MD (95%CI)	*P* value
Self-reported sleep parameters										
PSQI score	10.6 (0.62)	6.8 (0.62)	6.67 (0.62)	11 (0.76)	8 (0.76)	7.7 (0.76)	−0.8 (−3.14, 1.54)	0.502	−0.63 (−3.94, 2.67)	0.707
Self-reported sleep quality	1.73 (0.14)	1.0 (0.14)	0.93 (0.14)	1.5 (0.17)	1.2 (0.17)	1.6 (0.17)	−0.43 (−1.05, 0.18)	0.166	−0.9 (−1.77, −0.03)	0.042
Self-reported sleep latency (minutes)	38.33 (4.53)	20 (4.53)	17.67 (4.53)	30.5 (5.54)	20.5 (5.54)	10.9 (5.54)	−8.33 (−25.43, 8.77)	0.340	−1.07 (−25.25, 23.12)	0.931
Self-reported sleep duration (h)	5.33 (0.24)	5.97 (0.24)	5.97 (0.24)	4.7 (0.29)	5.35 (0.29)	5.55 (0.29)	−0.02 (−0.88, 0.85)	0.970	−0.22 (−1.43, 1.00)	0.727
Self-reported sleep efficiency (%)	74 (4)	83 (4)	82 (4)	68 (5)	82 (5)	83 (5)	−6 (−21, 9)	0.446	−7 (−29, 15)	0.550
Actigraphy sleep parameters										
Time in bed (h)	7.35 (0.22)	7.27 (0.22)	-	6.81 (0.27)	7.06 (0.27)	-	−0.33 (−0.97,0.30)	0.307	-	-
Sleep duration (h)	6.18 (0.20)	6.14 (0.20)	-	5.46 (0.24)	5.84 (0.24)	-	−0.41 (−1.10, 0.28)	0.246	-	-
Sleep efficiency (%)	84.54 (1.48)	84.43 (1.48)	-	80.53 (1.81)	82.58 (1.81)	-	−2.17 (−7.03, 2.70)	0.382	-	-
WASO (min)	43.76 (3.90)	44.06 (3.90)	-	42.35 (4.77)	37.39 (4.77)	-	5.26 (−5.18, 15.69)	0.324	-	-
Sleep latency (min)	12.92 (3.28)	14.20 (3.28)	-	23.06 (4.02)	20.30 (4.02)	-	4.04 (−4.18, 12.25)	0.335	-	-
Sleep midpoint (hh: mm)	02:39 (00:14)	02:42 (00:14)	-	02:02 (00:17)	02:14 (−00:17)	-	−9.0 min (−40, 23)	0.590	-	-
Sleep regularity (SD of sleep duration) (min)	60.95 (7.03)	50.30 (7.03)	-	54.61 (8.60)	75.66 (8.60)	-	−31.69 (−57.92, −5.47)	0.018	-	-

Values are presented as mean (standard error). Between-group differences represent mean differences (MD) with 95% confidence intervals

For metabolic parameters (Table 5), fasting glucose levels at week 8 were significantly lower in the CBTI group compared to the HE group, MD −34.27 mg/dL (95% CI: −55.16, −13.37; *P* = 0.001). A1C, insulin and hs-CRP levels did not differ between the two groups. Lastly, no significant differences were observed in patient-reported outcomes (Table 5).

**Table 5:** Per-protocol analysis of metabolic parameters and sleep/psychological questionnaires at baseline, week 8 and week (*N* = 25)

Response variables	CBTI (*n* = 15)			HE (*n* = 10)			Between-group difference in change from baseline (CBTI- HE)			
							Week 8 vs. baseline		Week 16 vs. baseline	
	Baseline	Week 8	Week 16	Baseline	Week 8	Week 16	MD (95%CI)	*P* value	MD (95%CI)	*P* value
Metabolic parameters										
Fasting glucose (mg/dL)	147.93 (7.83)	130.67 (7.83)	-	142.3 (9.59)	159.3 (9.59)	-	−34.27 (−55.16, −13.37)	0.001	-	-
A1C (%)	7.78 (0.23)	7.50 (0.23)	^−^	7.18 (0.28)	7.53 (0.28)	-	−0.63 (−1.35, 0.08)	0.082	-	-
Insulin (uIU/mL)	11.86 (2.25)	11.58 (2.25)	-	8.72 (2.76)	8.25 (2.76)	-	0.19 (−1.66, 2.03)	0.843	-	-
hs-CRP (mg/L)	3.98 (1.60)	5.30 (1.60)	-	2.31 (1.96)	3.1 (1.96)	-	0.52 (−3.35, 4.40)	0.791	-	-
Sleep and Psychological Questionnaires										
ISI	16.33 (0.82)	10.4 (0.82)	7.13 (0.82)	16.5 (1.00)	12.7 (1.00)	10.4 (1.00)	−2.13 (−5.65, 1.38)	0.234	−3.1 (−8.07, 1.87)	0.222
ESS	8.13 (0.91)	8.0 (0.91)	6.0 (0.91)	8.4 (1.12)	5.9 (1.12)	8.2 (1.12)	2.37 (−1.64, 6.38)	0.247	−1.93 (−7.60, 3.74)	0.504
GAD-7	6.53 (0.74)	4.53 (0.74)	3.73 (0.74)	4.8 (0.90)	4.1 (0.90)	4.5 (0.90)	−1.3 (−3.48, 0.88)	0.242	−2.5 (−5.58, 0.58)	0.111
PHQ-9	7.6 (0.93)	5.87 (0.93)	5.0 (0.93)	9.6 (1.14)	6.9 (1.14)	6.1 (1.14)	0.97 (−1.79, 3.72)	0.492	0.9 (−3.00, 4.80)	0.651
T-PSS-10	13.93 (1.26)	12.5 (1.26)	12.47 (1.26)	17.7 (1.54)	15 (1.54)	12.6 (1.54)	1.3 (−2.95, 5.55)	0.549	3.63 (−2.38, 9.65)	0.236
WHOQOL-BREF-THAI	91.67 (2.69)	98.33 (2.69)	98.67 (2.69)	89.4 (3.29)	90.5 (3.29)	96.0 (3.29)	5.57 (−1.37, 12.50)	0.116	0.4 (−9.41, 10.21)	0.936

Values are presented as mean (standard error). Between-group differences represent mean differences (MD) with 95% confidence intervals

*ISI* Insomnia Severity Index, *GAD-7* Generalized Anxiety Disorder Scale, *PHQ-9* Patient Health Questionnaire-9, *T-PSS-10* The Thai version of the Perceived Stress Scale-10, *WHOQOL-BREF-THAI* A Thai-language version of the WHOQOL-BREF, *ESS* The Epworth Sleepiness Scale

### Program satisfaction

Participants in both groups expressed similarly high satisfaction to the program, 4.42 ± 0.45 in the CBTI vs. 4.47 ± 0.44 in the HE group (*P* = 0.707).

### Adverse events

There were no adverse events reported during the study.

## Discussion

This study explored the effects of remotely-delivered CBTI in persons with T2D and insomnia. The results demonstrated that CBTI led to a more regular sleep as compared to health education, a difference in night-to-night variation of sleep duration of 21.8 min. At week 16, the CBTI group demonstrated less anxiety symptoms than the HE group. Furthermore, at week 8, the per-protocol analysis confirmed an increase in sleep regularity of 31 min in the CBTI as compared to the HE groups, and a clinically significant reduction in fasting glucose levels of 34 mg/dL, along with an improvement in self-reported sleep quality at week 16. These results demonstrated the benefits of remotely delivered CBTI in persons with T2D and insomnia on sleep and glycemic control, and also highlighted that program adherence is essential in achieving these benefits.

To date, only a few studies have explored the benefits of CBTI in persons with T2D and insomnia. In a study of 57 participants [25], *I-Sleep* intervention, a five-week online CBTI e-health program completed by the participants at home, did not result in improved glycemic control or objective sleep parameters as compared to usual care group. However, the CBTI participants had improved insomnia and depressive symptoms. Low adherence might have affected the results as only half of the participants completed the program [25]. This result was in contrast to our study which also utilized the online platform but the sessions were led by the researchers, likely resulting in a greater adherence. Two additional RCTs (*n* = 28 and *n* = 187) [9, 10] involving persons with T2D and insomnia symptoms or poor sleep quality revealed that in-person CBTI sessions led to an improvement in glycemic control (A1C 0.5–0.89%), diabetes-self-care behaviors, self-reported sleep quality and quality of life as compared to the control groups. Similarly to our study, adherence was crucial as there was a significant association between the number of days participating in the CBTI and improved blood glucose levels [10]. Another single-arm prospective study in eight post-menopausal women with T2D and insomnia showed that an 11-week CBTI improved PSQI scores and reduced insomnia symptoms at the 3-week follow-up, while there was no significant change in A1C [26]. Our study demonstrated increased in objectively measured sleep regularity as a result of CBTI which has not been previously reported in T2D population. Irregular sleep has recently been reported to be associated with various cardiometabolic outcomes including metabolic syndrome, systemic inflammation and poor glycemic control [27]. Therefore, a more regular sleep could be associated with improved metabolic health. In the current study, self-reported sleep quality (item 6 of the PSQI) improved in the CBTI group but the global PSQI score and ISI did not differ between groups. This was likely due to a similar reduction in PSQI and ISI in both groups after the intervention, resulting in non-significant differences between groups. It is possible that participating in group sessions, such as HE, could lead to improved sleep. These results highlighted the importance of having an attention control group, instead of usual care, to differentiate the true effects of CBTI on outcomes.

Besides CBTI, several studies have shown the benefits of sleep hygiene education in persons with T2D [28–30] including improved self-reported sleep quality [28–30], diabetes distress [30], and reduced hospital visits [29] while the results on glycemic control differed [28, 29]. A meta-analysis of 11 studies using either CBTI or sleep education/hygiene in T2D showed that sleep quality improved and A1C reduced by 0.43% [31]. Collectively, these data, along with the results of the current study, suggested that CBTI and behavioral sleep interventions in persons with T2D, delivered remotely or in-person, lead to improved subjective and objective sleep parameters as well as glycemic control.

There are possible several mechanisms by which poor sleep quality, fragmented sleep and insomnia symptoms contribute to poor glycemic control. Sleep disturbances and insomnia can increase inflammatory cytokines (hs-CRP, tumor necrosis factor, interleukin 6) and enhance the hypothalamic-pituitary-adrenal activity, leading to reduced β-cell function, increased insulin resistance, and contributing to hyperglycemia [32]. Insomnia also often coexists with depression [33], both of which can negatively impact diabetes self-care behaviors (e.g. medication adherence, physical activity, dietary habits), resulting in poorer glycemic outcomes. Interventions targeting both sleep quality and mental health may enhance metabolic outcomes and overall well-being.

Our study had several strengths including the utilization of comprehensive sleep assessments (subjectively and objectively), and objective evaluation of glycemic control and inflammation. The inclusion of the HE group which received equal time from the researchers as the CBTI group facilitated the interpretation of the CBTI effects from attention effects. Confounding effects of diabetes medication on glycemic control were also minimized as participants were on a stable regimen throughout the study. The study also confirmed the efficacy of remotely-delivered CBTI in persons with T2D. There were, however, a few Limitations. Factors such as changes in dietary patterns or physical activity, which could influence sleep quality and glycemic parameters, were not collected. In addition, only subjective sleep parameters were captured at week 16. The objective sleep parameters at that time could have provided information of CBTI durability on objective sleep measures. Lastly, glycemic control improved only in those who were adherent to the protocol, suggesting that strategies to enhance adherence should be incorporated in the intervention design.

In conclusion, remotely-delivered CBTI was superior to health education in improving sleep regularity and reducing anxiety symptoms in T2D with insomnia. Adherence to CBTI led to significantly reduced fasting glucose, supporting its role in glycemic management. Sleep assessment and tailored insomnia interventions such as CBTI should be integrated into T2D standard care to optimize metabolic and sleep outcomes.

## Supplementary Information

Below is the link to the electronic supplementary material.

Supplementary Material 1

## Data Availability

The data that support this study are available from the corresponding author upon reasonable request.

## Declarations

### Clinical trial registration

clinicaltrials.gov, NCT06202742 (12/12/2023).
